# Development and Validation of a Prechiasmatic Mouse Model of Subarachnoid Hemorrhage to Measure Long‐Term Cognitive Deficits

**DOI:** 10.1002/advs.202403977

**Published:** 2024-10-23

**Authors:** Deepti Diwan, Jogender Mehla, James W. Nelson, James D. Quirk, Sheng‐Kwei Song, Sarah Cao, Benjamin Meron, Aminah Mostofa, Gregory J. Zipfel

**Affiliations:** ^1^ Department of Neurological Surgery Washington University School of Medicine St. Louis MO 63110 USA; ^2^ Mallinckrodt Institute of Radiology Washington University School of Medicine St. Louis MO 63110 USA

**Keywords:** long‐term cognitive impairment, prechiasmatic cistern, subarachnoid hemorrhage

## Abstract

Controllable and reproducible animal models of aneurysmal subarachnoid hemorrhage (SAH) are crucial for the systematic study of the pathophysiology and treatment of this debilitating condition. However, current animal models have not been successful in replicating the pathology and disabilities seen in SAH patients, especially the long‐term neurocognitive deficits that affect the survivor's quality of life. Therefore, there is an unmet need to develop experimental models that reliably replicate the long‐term clinical ramifications of SAH – especially in mice where genetic manipulations are straightforward and readily available. To address this need, a standardized mouse SAH model is developed that reproducibly produced significant and trackable long‐term cognitive deficits. SAH is induced by performing double blood injections into the prechiasmatic cistern – a simple modification to the well‐characterized single prechiasmatic injection mouse model of SAH. Following SAH, mice recapitulated key characteristics of SAH patients, including cerebral edema measured by MRI ‐ an indicator of early brain injury (EBI), neuroinflammation, apoptosis, and long‐term cognitive impairment. This newly developed SAH mouse model is considered an ideal paradigm for investigating the complex SAH pathophysiology and identifying novel druggable therapeutic targets for treating SAH severity and SAH‐associated long‐term neurocognitive deficits in patients.

## Graphical abstract

This study presents a novel prechiasmatic mouse model of subarachnoid hemorrhage (SAH) through a double blood injection. This model provides a reliable and reproducible approach for assessing long‐term neurocognitive deficits after SAH and holds promise for identifying potential therapeutic targets to alleviate SAH‐induced long‐term neurological cognitive impairments in patients.

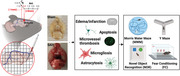

## 1 Introduction

Spontaneous subarachnoid hemorrhage (SAH) from rupture of an intracranial aneurysm leads to significant mortality and long‐term neurological deficits, with ≈50% of SAH patients suffering major cognitive deficits precluding their return to work or resumption of daily activities.^[^ ^1^, ^2^, ^3^ ^]^ Several methods for modeling SAH have been developed in an effort to provide an experimental foundation for mechanistic and therapeutic investigation in the preclinical setting. Most of these animal models recapitulate some, but not all, of the acute pathophysiological events that occur after SAH, including the development of secondary brain injury from Early Brain Injury (EBI) and Delayed Cerebral Ischemia (DCI).^[^ ^4^ ^]^ The long‐term consequences of SAH are less well‐characterized, especially its deleterious impact on cognitive function.

The most frequently used animal models of SAH are: i) Endovascular perforation model, which involves advancing a suture into the internal carotid artery (ICA) until it perforates a vessel within the Circle of Willis, leading to extravasation of blood into the basal cisterns;^[^ ^5^, ^6^, ^7^, ^8^, ^9^ ^]^ and ii) Direct injection of blood into the cisterna magna or prechiasmatic cistern,^[^ ^10^, ^11^, ^12^ ^]^ where the former results in a blood clot primarily localized around vessels of the posterior circulation and the latter around vessels of the anterior circulation.^[^ ^13^, ^14^ ^]^ Each of these models has its own unique set of advantages and disadvantages, but none fully recreates the pathologic conditions of human SAH.^[^ ^15^, ^16^, ^17^ ^]^ One of the many reasons for this situation could be that rodent models of SAH are technically demanding and have been difficult to standardize for variability in hemorrhage severity.^[^ ^18^ ^]^

Regarding long‐term cognitive deficits after experimental SAH, a variety of induction methods, rodent species, and time points have been examined. To date, the most consistent and trackable neurocognitive deficits have been demonstrated in rat models of SAH, including endovascular perforation,^[^ ^19^ ^]^ prechiasmatic blood injection,^[^ ^20^ ^]^ and cisterna magna blood injection^[^ ^20^, ^21^ ^]^ techniques. All three induction methods produce significant neurocognitive deficits in rats up to 1‐month after ictus. However, rats are more expensive than mice, and targeted genetic manipulation is significantly more challenging. In contrast, long‐term neurocognitive deficits in SAH mouse models have proven significantly more challenging. For prechiasmatic and cisterna magna blood injections mouse models, neurocognitive deficits have only been demonstrated up to 2 weeks after ictus (**Table** **1**).^[^ ^12^, ^22^, ^23^ ^]^ For the endovascular perforation mouse model, disparate results have been reported with Regnier‐Golanov et al. reporting significant neurocognitive deficits 1‐month after ictus using a battery of behavioral tests including Morris Water Maze,^[^ ^24^ ^]^ while Milner et al. showed no consistent neurocognitive deficits 1‐month after ictus as assessed by Morris Water Maze^[^ ^25^ ^]^ and Fanizzi et al. showed minimal long‐term neurobehavioral deficits 1‐month after ictus using a battery of behavioral tests including Morris Water Maze.^[^ ^26^ ^]^ The development of a mouse model of SAH that produces robust and consistent long‐term neurocognitive deficits is highly desirable, as it would accelerate experiments, lower costs, and leverage the tremendous power of genetically manipulated mice.

**Table 1:** Comparison with existing SAH mouse models.

	Double prechiasmatic injection mouse model	Single prechiasmatic injection mouse models				Endovascular perforation mouse models			Cisterna magna injection mouse models		
	Current Study	Sabri et al. 2009	Boettinger et al. 2017		Chung et al. 2020	Milner et al. 2014	Fanizzi et al. 2017	Regnier‐Golanov et al. 2021	Kamp et al. 2014	Turan et al. 2020	Pedard et al. 2020
Strain	C57BL/6J	CD1	C57BL/6J		C57BL/6J	C57BL/6J	C57BL/6J	C57BL/6J	C57BL/6J	C57BL/6J	C57BL/6J
Age of mIce (weeks)	12–14	10–12	10–12		10–12	12–14	12	10–14	15–20	6	8–10
SAH induction technique	*First injection*: 100 µl blood or aCSF *Second injecction (24 h apart)*: 100 µl blood or aCSF	Single injection: 100 µl blood or Saline	Single injection:80 µl, 100 µl, 120 µl blood or aCSF		Single injection: 80 µl blood or Saline	SAH = Suture perforation at ICA bifurcation; Sham = Suture introduction without perforation	SAH = Suture perforation at ICA bifurcation; Sham = Suture introduction without perforation	SAH = Suture perforation at ICA bifurcation; Sham = Suture introduction without perforation	Single injection: 50 µl blood or Saline	Single injection: 60 µl blood or Saline	*First injection*: 60 µl blood or aCSF *Second injection (24 h apart)*: 30 µl blood or aCSF
*Behavioral Tests and Assessment Timepoint*											
Neuroscore			2 d **	7 d		1 d ‐ 3 d **	1, 3, and 7 d **	4 d	3 d **	2 d	
Morris Water Maze	30 d **				30 and 90 d	23 d	24 d			16 d	
Y‐maze	30 d **				30 and 90 d **			4 d **			
Novel Object Recognition	30 d **							30 d **			
Fear Conditioning	30 d **										
Elevated Plus Maze							27 d			16 d	
Barnes Maze								30 d **			
Social Interaction							29 d	4 d **			
Rotarod					30 and 90 d		7 d				
Open Field Test					30 and 90 d		27 d **	30 d **			9 d **
Beam Balance Test			2 d **	7 d							10 d **
cccccccFlex Field			2 d **	7 d							
SHIPRA Score			2 d **	7 d							
*Biochemical Markers*											
Microgliosis (Iba‐1)	30 d **									16 d	
Astrogliosis (GFAP)	30 d **		2 d **	7 d							
TUNEL Assay		7 d **	2 d **	7 d							
Fluoro‐jade		7 d **	2 d **	7 d						16 d	
Fibrinogen	30 d **		2 d **	7 d							
Nissl‐staining						23 d					

** denotes statistical significance.

To address this important investigational gap, we developed a “prechiasmatic double injection” mouse model of SAH in which heterologous arterial blood is injected twice into the prechiasmatic cistern 24 h apart. In the short term, the model was assessed for prototypic SAH‐related brain injury, including EBI, which was identified by the presence of cerebral edema via MRI and the development of blood‐brain barrier disruption, apoptosis, and neuroinflammatory changes. In the long term, the model was validated using a series of well‐characterized behavioral tests to assess long‐term neurocognitive deficits 30 days after SAH.

## 2 Experimental Section

### 2.1 Animals

All experimental procedures were carried out following the guidelines for the care and use of animals in research, which were established by the Institutional Animal Care and Use Committee at Washington University and approved by the National Institutes of Health (Protocol ID: 24–0126). Male C57BL/6J mice between 12–14 weeks old were obtained from Jackson Laboratories (Bar Harbor, ME). The mice were provided ad libitum access to water and a standard mouse chow dietand were acclimated to a controlled environment with a 12 h light/dark cycle. Animals used in this study underwent pre‐screening using neuroscore as per the established protocol.^[^ ^6^, ^7^, ^27^ ^]^ Briefly, neurological function was graded based on the combination of a motor score (0–12) that evaluated spontaneous activity, symmetry of limb movements, climbing, balance, and coordination, and a sensory score (4–12) that evaluated body proprioception and vibrissae, visual, and tactile responses (data not shown). The animals were randomly assigned to experimental groups. All the experiments were performed by a blinded observer.

### 2.2 SAH Induction in Mice

**Figure 1:**
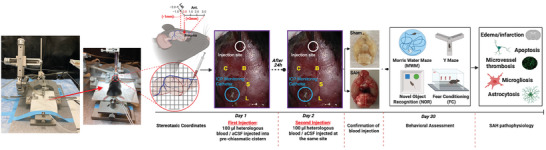
Overview of the protocol for developing and assessing prechiasmatic double blood injection mouse model of subarachnoid hemorrhage (SAH). The protocol includes preparation, pre‐ and post‐procedure management of animals, behavioral assessments at certain time points post‐SAH, and finally, euthanasia and injury‐induced thromboinflammation analysis.

Experimental SAH was induced in mice using the prechiasmatic injection technique (**Figure** **1**). Briefly, the mice were first anesthetized with isoflurane (2% induction, 1.5% maintenance) in a mixture of 70:30 N_2_O:O_2_, and their body temperature was maintained at 37 °C with a rectal temperature probe and a thermo‐regulated heating pad. The mice were then secured in a stereotaxic frame, and the scalp was incised to expose the surface of the dorsal skull. An intracranial pressure (ICP) probe was placed to monitor ICP during and after the procedure. A hole was made in the atlanto‐occipital membrane using a 23G needle to insert the ICP probe. The probe was pulled slightly to ensure that it showed a pulsating curve ranging between 0–5 mmHg.

For SAH induction, an incision was made in the midline of the anterior scalp, exposing the transparent skull. The superior sagittal sinus was observed and avoided during SAH induction. A 0.7 mm burr hole was drilled in the skull 5 mm anterior to the bregma and slightly off the midline to avoid the sagittal sinus. At this point, 0.1 ml of fresh heparinized blood was collected from a littermate donor. The heterologous blood was used for SAH induction because autologous blood collection was associated with a variety of potential complications and concerns, including altered hemodynamics due to blood loss, added animal stress, and increased anesthetic time. Consequently, heterologous blood was routinely used to induce experimental SAH.^[^ ^11^, ^28^ ^]^ A 27G spinal needle connected to a 1cc syringe was positioned with the side port facing up and angled 32° from the vertical axis. The needle was advanced carefully until contact was made with bone at a depth of ≈7 mm, and then the needle was retracted 0.5–1.0 mm. Hundred microliters of blood for SAH induction or artificial cerebrospinal fluid (CSF) for sham controls was injected through the spinal needle over 40–45 s using a digital infusion pump. The needle was kept in place for an additional 2–3 min to prevent backflow of injected blood or CSF leakage, then slowly withdrawn. The second blood injection was performed at the same site 24 h after the first injection using the same protocol (Figure 1). Following surgery, any residual blood products or debris were cleared from the skull surface. The drilling point was sealed with bone wax, and the incision was closed with a sterile suture. The mice were allowed to recover from anesthesia in an incubator and then returned to their home cages.

In the study, 42 animals were utilized. Following the induction of SAH, eight animals died, resulting in an overall mortality rate of ≈20%. The death of the animals occurred either during the experimental procedure or within 24 h post‐surgery, primarily due to weight loss.

Mice were subjected to transcardial perfusion with heparinized PBS, and the brain was carefully removed to examine the blood distribution along subarachnoid vessels. No visible blood was observed in the sham‐operated animals. In contrast, in the case of SAH, extravasated blood was consistently found covering the middle cerebral artery (MCA) and distributed along larger arteries that branch from the MCA. Any animals that died during or immediately after the surgical procedure, those from the SAH group without subarachnoid blood, those from the sham group with subarachnoid blood, and those with severe hemiparesis within 6 h of surgery (indicative of inadvertent MCA occlusion) were excluded from the analysis. All sham and SAH‐operated animals that survived and were able to complete neurobehavioral assessments were included in the analysis.

### 2.3 MRI Protocol

**Figure 2:**
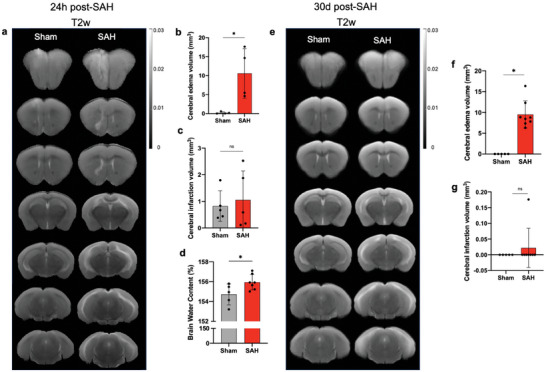
Cerebral edema and cerebral infarction assessment in prechiasmatic mouse model of SAH. MRI for mice a,e). Regions of edema are bright on T2‐weighted images for the SAH group vs. sham controls. Quantification revealed significantly large edema volumes b,f) and no infarct c,g) volumes in SAH group compared to sham controls. Data show means ± SD. Student's t‐test. *p < 0.05; vs. sham group; *n* = 5 per group. In a separate experiment, WT mice underwent SAH or sham surgery. Brain water content was measured 24 h after SAH induction d). Data show means ± SD. Student's t‐test. *p < 0.05; vs. sham group. *N* = 5 per group.

All in vivo MRIs were conducted using a Bruker BioSpec 9.4 T MRI (Bruker, Ettlingen, Germany) using a 4‐channel mouse brain receiver coil in combination with a quadrature transmit coil (**Figure** **2**).^[^ ^29^, ^30^ ^]^ Mice were anesthetized using 1–1.5% isoflurane/O_2_ and secured in the prone position. Respiratory rate and rectal temperature were monitored using a small‐animal physiological monitoring system (SA Instruments; Stony Brook, NY) and mouse body temperature was maintained at 37 °C using a circulating warm water pad.

Axial T2‐weighted fast spin echo anatomic images with fat suppression were acquired at 63 × 63 × 500 µm^3^ resolution using the following parameters: repetition time (TR) = 2750 ms, echo time (TE) = 35 ms, 6 signal averages, rare factor = 8. Echo‐planar imaging (EPI) Diffusion tensor images were acquired at 167 × 167 × 500 µm^3^ resolution using the following parameters: TR = 2000 ms, TE = 24 ms, 2 averages, δ = 3 ms, Δ = 10 ms, 98 diffusion directions spread over 11 shells with a maximum b‐value of 1500 s mm^−2^ and 8 additional b = 0 images.

### 2.4 Brain Water Content

Brain water content was measured 24 h after SAH induction. It was calculated by weighing brains immediately (wet weight) and after drying at 80 °C for 24 h (dry weight). The percentage of water content was calculated as [(wet weight – dry weight)/wet weight] × 100%.^[^ ^31^ ^]^

### 2.5 Behavioral Assessment Methods

#### 2.5.1 Morris Water Maze

The Morris water maze was performed in accordance with the previous studies (Figure 6).^[^ ^32^, ^33^, ^34^ ^]^ Briefly, two principal axes of the maze were the standard designation; with each line bisecting the maze was perpendicular to the another, dividing it into four imaginary cardinal poles: North (N) – opposite point of the experimenter, South (S) – experimenter's position, east (E) – experimenter's right and West (W) – experimenter's left; thereby creating four quadrants.

A pool filled with opaque, non‐toxic white paint was used for all experiments. An escape platform was placed in one of the quadrants of the pool beneath the water surface (0.5–1 cm), located halfway between the wall and the center of the pool. The water temperature was maintained at 22 ± 1 °C to prevent the mouse from floating. Three cues of different shapes were placed around the water pool to help the mouse in finding the platform. Mice were randomly placed in the water, and an automated ANY‐Maze Behavioral Tracking Software (Stoelting) was used to record the time taken to reach the escape platform (escape latency) and swim speed. The mice underwent four trials per day at each of the four cardinal drop points (north, south, east, west) in random order for eight consecutive days, with the platform hidden at the same location each day. If the mice failed to locate the escape platform within the 60 s trial length, mice were manually guided to the platform and kept there for 15 s. A single probe trial without a hidden platform was completed on the 9th day. Data was analyzed using Any‐Maze behavioral tracking software.

#### 2.5.2 Y‐Maze

The Y‐maze test is a method used to evaluate spatial working and reference memory in rodents, as described in previous studies.^[^ ^32^, ^33^, ^34^ ^]^ Briefly, The test involves a Y‐shaped maze consisting of three white, opaque plastic arms at a 120° angle. The mice were placed in the center of the maze and allowed to explore two arms for 10 min during the training trial. The third arm (novel arm) was kept closed (Figure 7). After a 30 min inter‐trial interval, the blocked arm was opened, and the mice were placed again in the center of the maze to explore all three arms for 5 min during the test trial. Over the course of multiple arm entries, the animal should display a tendency to enter the third arm that was less visited. An entry was defined as when all four limbs were within the arm, and the time spent in novel arm entries by mice was then calculated. Data was analyzed using Any‐Maze behavioral tracking software.

#### 2.5.3 Novel Object Recognition (NOR)

The NOR test is a widely used method for assessing the memory of rodents. The test is based on the innate inquisitiveness of rodents to explore novel objects and retain their spatial memory.^[^ ^33^, ^34^, ^35^, ^36^ ^]^ The test was conducted in an open‐field arena, to which the mice were first habituated. On the next day, the mice were then introduced into the arena with two identical objects and allowed to explore for 10 min. The mice were then removed, and one of the two identical objects was replaced with a novel object. Following a 30 min inter‐trial period, the mice were returned to the arena, and their exploration behavior for novel objects was recorded using a video camera for 5 min. The mice were considered to be engaged in object exploration when their head was oriented within 45° of an object and within 4 cm of it. Rearing with the head oriented upward was also included if at least one forepaw was on the object. However, climbing over or sitting on the objects was not included. Finally, the exploration ratio was calculated.

#### 2.5.4 Fear Conditioning (FC)

FC test was conducted to evaluate amygdala‐ and hippocampus‐dependent memory in rodents, as described in the previous publications.^[^ ^32^, ^34^, ^36^ ^]^ The test was carried out in an acrylic square chamber (33 × 33 × 25 cm) with a stainless steel rod floor that was connected to a shock generator for delivering a footshock. A tone stimulus was delivered through a speaker. Before conditioning, the chamber was cleaned using a 1% Virkon solution to mask any former odor cues. On the day of conditioning, mice were transported from their home cage to a testing room and allowed to sit undisturbed in their cages for 10 min. Mice were then placed in the conditioning chamber and allowed to explore for 2 min before the onset of the tone (20 s, 2000 Hz). In the delay conditioning procedure, a 2 s and 0.5 mA shock was given in the last 2 s of the tone duration. Mice recieved five delayed conditioning trials, with 120 s intertrial intervals (ITI). One minute after the last shock, the mice were removed from the conditioning chamber and returned to their home cages. After 24 h, the tone test was conducted in a triangular chamber situated in a different room. The chamber was geometrically different from the conditioning chamber, thereby enabling the assessment of the tone conditioning in the absence of the training context. The chamber was cleansed with 70% isopropyl solution after every mouse. During the tone test, three 20 s tones were given after a 2 min baseline period, with each tone separated by a 120 s ITI. The mice were removed from the triangular chamber 1 min after the last tone presentation and returned to their home cages. The freezing response was measured using a time sampling procedure, in which an observer scored the presence or absence of the freezing response for each mouse at every 2 s interval. Twenty‐four hours after the tone test, a context test was conducted by placing each mouse back in the original conditioning chamber for 5 min. During this test, freezing was scored for each mouse at every 5 s interval. Percent freezing was calculated using Any‐Maze behavioral tracking software.

### 2.6 TUNEL Assay

Apoptosis was detected using an in situ cell death detection kit (fluorescein; Roche). Terminal deoxynucleotidyl transferase dUTP nick end labeling (TUNEL) was performed on 40 µm coronal sections following the manufacturer's protocol. The slides were then counter‐stained with 4′,6‐diamidino‐2‐ phenylindole (DAPI), washed, and coverslipped with a Vectashield® antifade mounting medium, and sealed with nail polish. Finally, the samples were imaged using a nanozoomer, and the images were analyzed with Image‐J software. Three to five brain sections per animal were analyzed in each group.

### 2.7 Western Blot Analysis

A Western blot analysis was performed following previously established procedures.^[^ ^6^, ^33^ ^]^ The brain samples were homogenized using an ultrasonic homogenizer in a lysis buffer containing protease and phosphatase inhibitor cocktail (PI78441, Thermo Scientific). After centrifugation, the supernatant was transferred to a new tube. The protein concentration was measured using the BCA Protein Assay Kit (5000112; BioRad)and protein was separated using Bolt™ Bis‐Tris Plus Mini Protein (NW04120BOX; Invitrogen) precast polyacrylamide gels. Then proteins were transferred to nitrocellulose membranes using iBlot™3 transfer stacks (IB33002, Invitrogen).

The membranes were blocked with 5% nonfat milk in Tris‐buffered saline and Tween 20 (TBST) at pH 7.6 and were then incubated overnight with primary antibodies: anti‐TNF‐alpha (1:1500, catalog number T8300, sigma‐Aldrich); Caspase‐3 (1:1500, 9662, Cell Signaling Technology); anti‐claudin‐5 (1 : 500, 352500; Thermo Fisher), anti‐β‐actin (1:5000), and anti‐β‐tubulin (1 :5000, Abcam). Subsequent to three washes with TBST, the membranes were exposed to horseradish peroxidase‐conjugated anti‐IgG secondary antibodies for 1.5 h at room temperature, followed by washing with TBST three times for 15 min each. The membranes were incubated with an enhanced chemiluminescence buffer (32106; Thermo) and visualized using the Bio‐Rad imaging system (Bio‐Rad, Hercules, CA, USA).

### 2.8 Immunohistochemistry

Immunohistochemistry was performed as previously described.^[^ ^6^, ^8^, ^32^, ^33^, ^34^, ^37^ ^]^ Free‐floating brain sections with a thickness of 40‐µm were subjected to fluorescent immunohistochemical staining using the following primary antibodies: rabbit anti‐fibrinogen (ab34269, 1:1000 dilution), mouse anti‐Iba1 monoclonal antibody (MA5‐27726, 1:1000 dilution), and mouse anti‐glial acidic fibrillary protein (GFAP) (IF03L, 1:1000 dilution). The secondary antibodies used included ant‐rabbit Alexa Fluor 488, and anti‐mouse Alexa Flour 594. In brief, free‐floating brain sections were washed with TBS and then blocked in TBS containing 0.3% Triton‐X 100 and 3% goat serum for 2 h. Subsequently, the brain sections were incubated in primary antibodies while being shaken at room temperature for 24 h. Three 10 min washes were performed after 24 h, and the brain sections were incubated in secondary antibodies for another 24 h. After incubation, the brain sections were washed three times for 10 min each. Next, brain sections were then mounted on slides using Vectashield H‐1000 (Vector Laboratory), and the slides were sealed with nail polish. Finally, the samples were imaged using a nanozoomer, and the images were analyzed with Image‐J software.

### 2.9 Statistical Analysis

Statistical analysis was performed using GraphPad Prism 9.0 (GraphPad Software Inc., CA, USA). Sample sizes were estimated using effect sizes based on the previous studies^[^ ^32^, ^33^, ^34^, ^36^ ^]^ and pilot experiments. An independent two‐tailed t‐test was used to obtain statistically significant differences between the experimental groups. A one‐way repeated measures ANOVA was used to measure the significant differences in escape latency and swim speed across the days during the acquisition phase of MWM. All data were tested for normality using the Shapiro‐Wilk test. Results represented as mean ± SD. Statistical significance was set at *P* < 0.05.

## 3 Results

A standardized rodent model that closely replicates human pathophysiology of SAH, including reliable and trackable long‐term neurocognitive deficits, is important to develop for use in preclinical studies. In this context, a new and improved version of the previously described prechiasmatic blood injection mouse model of SAH is presented – referred to as the “double prechiasmatic injection” mouse model of SAH. The model entails two discrete injections (24 h apart) of determined volumes of heterologous whole blood into the prechiasmatic cistern. This SAH model has a distinct advantage over currently used experimental mouse models of SAH in that it causes highly reproducible injury severity, leading to robust neurocognitive deficits 1‐month after ictus, along with trackable chronic neuroinflammatory changes. The prechiasmatic double injection model eliminates possible confounding variables by allowing control over the initiation, volume, and rate of hemorrhage. It also allows for variation of blood volumes and injection rates to optimize resulting neurocognitive deficits across mouse strains, conditions, and laboratories.

### 3.1 EBI Assessment

SAH‐induced EBI is intricately linked to an unfavorable patients outcome. We found a substantial increase in brain water content 24 h following SAH (Figure 2d). We also confirmed the presence of cerebral edema 24 h and 30 days after SAH using MRI (Figure 2). Specifically, significantly elevated signals, indicative of space‐occupying cytotoxic edema after SAH, were noted in the T2‐weighted images within the hemispheres of SAH mice compared to the sham group (Figure 2a,b), which is consistent with our brain water content data (Figure 2d). Our results show no significant difference in the extent of infarction between SAH and sham animals, indicating that our model is primarily one of SAH‐induced brain injury (Figure 2c).

### 3.2 SAH‐Induced Pathophysiology

**Figure 3:**
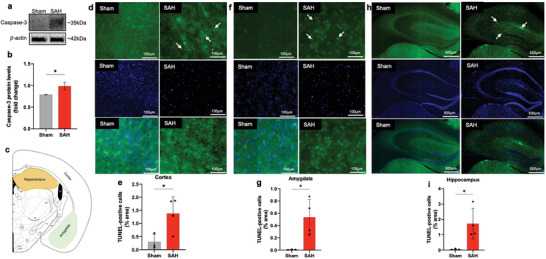
SAH‐induced apoptosis in mice. Immunoblots and quantification of caspase‐3 expression in ipsilateral mice brain post‐SAH assessed by densitometric analysis normalized by 𝛽‐actin protein levels and expressed as fold change compared to the sham‐operated animals a,b). Representative demarcation of analyzed areas for TUNEL‐positive cells c). Representative images and quantification of TUNEL‐positive cells in the cortical d,e), amygdala f,g), and hippocampus (h,i) sections from sham and SAH animals. Scale bars represent 100µm for the cortex and amygdala and 500µm for the hippocampus. Data show means ± SD. Student's t‐test., *p < 0.05; vs. sham group. *N* = 3–4 per group. Three to five brain sections per animal were analyzed in each group.

**Figure 4:**
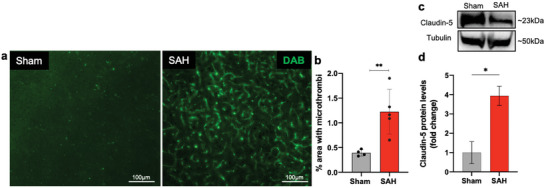
SAH‐induced microthrombosis in the delayed phase of the injury. Representative images of the fibrinogen‐positive microthrombi in mice that underwent sham and SAH surgery a). Brain tissue microthrombosis was determined as percent coverage of ipsilateral parietal cortex b). Immunoblots and quantification of claudin‐5 expression in ipsilateral mice brain post‐SAH assessed by densitometric analysis normalized by tubulin protein levels and expressed as fold change compared to the sham‐operated animals c,d). Scale bars represent 100 µm for the brain section. Data show means ± SD. Student's t‐test., *p < 0.05, **p < 0.01; vs. sham group. *N* = 4 per group. Three to five brain sections per animal were analyzed in each group.

**Figure 5:**
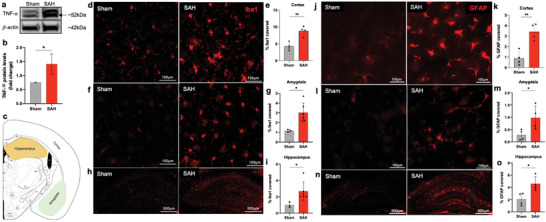
SAH‐induced sustained neuroinflammation in the delayed phase of the injury. Immunoblots and quantification of TNF‐𝛼 expression in ipsilateral mice brain post‐SAH assessed by densitometric analysis normalized by 𝛽‐actin protein levels and expressed as fold change compared to the sham‐operated animals a,b). Representative demarcation of analyzed areas c). The mice brain sections were immunostained with Iba‐1 and quantified for microgliosis in brain cortex d,e), amygdala f,g), and hippocampus h, i); and GFAP for astrocytosis in brain cortex j,k), amygdala l,m), and hippocampus n,o). Scale bars represent 100µm for the cortex and amygdala and 500µm for the hippocampus. Data show means ± SD. Student's t‐test., *p < 0.05; **p < 0.01; vs. sham group; *N* = 3–4 per group. Three to five brain sections per animal were analyzed in each group.

**Figure 6:**
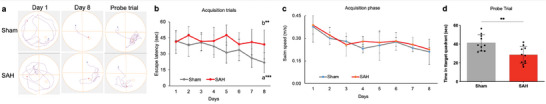
The SAH led to impaired spatial learning and memory in the Morris water maze. C57Bl/6J mice underwent SAH or sham. a) Representative paths of the two groups of mice. b) Escape latency during the acquisition phase. c) Swim speed during the acquisition phase. d) Time spent by each group in the target quadrant during the probe trial. Data show means ± SD. Student's t‐test. **p < 0.01; ***p < 0.001 vs. sham group. *N* = 5 per group; a‐as compared with day 1 for sham mice; b‐as compared with sham on day 8.

**Figure 7:**
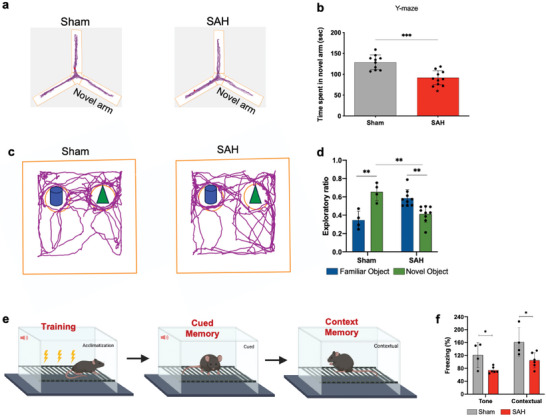
SAH induces behavioral deficits in prechiasmatic double blood injection mouse SAH model. WT mice underwent SAH or sham surgery and were evaluated 30 d post‐SAH for cognitive function. a) Animal movement traces of sham (Left) and SAH (Right) mice during the test phase. b) Novel arm entries in the Y‐maze test. c) Representative preferential investigation track of sham (Left) SAH (Right) mice for the novel objects. d) Exploratory ratio for familiar and novel objects. e) Mice were trained with fear conditioning and tested with their freezing responses to tone and context. f) Percent freezing of mice during tone and contextual fear conditioning test. Comparison of percentage of freezing of SAH mice with sham controls during tone and contextual fear conditioning tests. Data show means ± SD. Student's t‐test. *p < 0.05; **p < 0.01 vs. sham group. *N* = 5–10 per group.

The SAH‐associated inflammation are described well in the acute and subacute phases but haven't been reported in the delayed phase following injury to date, nor has it been evaluated for concurrence with neuroinflammation and delayed neurological deterioration. We are the first to establish this correlation of SAH insult in our prechiasmatic double blood injection mouse model of SAH (Figures 2, 3, 4, 5, 6, 7).

Claudins play a vital role in maintaining the integrity of brain blood vessels as the major proteins of the BBB.^[^ ^38^, ^39^ ^]^ While claudin‐1, claudin‐3, and claudin‐12 are expressed in the endothelial cells of the BBB, claudin‐5 is the predominant claudin in these cells and is considered the key factor in endothelial permeability at the BBB. In SAH, there is an increase in BBB permeability and a reduction in claudin‐5 expression levels in the brain. Western blotting results reinforce these observations, indicating a decrease in claudin‐5 protein expression in SAH mice compared to sham controls (Figure 4c,d).

The expression levels of the pro‐apoptotic protein caspase‐3 were found to be elevated in mice with SAH compared to the sham group, as illustrated in **Figure** **3**a,b. The results of the terminal deoxynucleotidyl transferase dUTP nick end labeling (TUNEL) assay demonstrated an increased presence of apoptotic cells in the cerebral cortex (Figure 3d,e), amygdala (Figure 3f,g), and hippocampus (Figure 3h,i) of the SAH cohort. In contrast, a lower proportion of apoptotic cells was observed in the sham‐operated group.

Several groups have reported a noteworthy association between neuroinflammation and microvascular thrombosis after experimental SAH.^[^ ^6^, ^40^, ^41^, ^42^ ^]^ This association aligns with clinical observations and is posited to contribute to secondary brain injury and neurological deterioration. Consistent with these findings, our prechiasmatic double injection model of SAH revealed a significant increase in fibrinogen, a marker for microvascular clots, 30 days after SAH compared to the control group without SAH (**Figure** **4**a,b). Our findings indicate a close correlation between the number of microvascular thrombi with SAH‐induced neurobehavioral deficits (Figures 6 and 7) and sustained neuroinflammation (**Figure** **5**) in SAH mice relative to the sham group. Moreover, we showed increased expression of pro‐inflammatory cytokines, TNF‐α (Figure 5a,b), microgliosis (Figure 5d–i), and astrocytosis (Figure 5j–o) in the different regions of the SAH brain at day 30 after injury, indicating the sustained post‐ictus influence on histopathological and neurological outcomes.

### 3.3 Long‐Term Neurobehavioral Impairment

In this mouse model of SAH, a battery of different neurobehavioural paradigms, such as Morris water maze (MWM), Y‐maze, novel object recognition (NOR), and fear conditioning (FC) were used for the assessment of long‐term neurobehavioural functions. Sham mice showed a statistically significant (*p* < 0.001) lower latency on day 8 as compared to day 1, indicating intact learning ability of these mice (Figure 6b). In contrast, SAH mice showed impairment in learning behaviour as indicated by a non‐significant difference in the escape latency on day 8 in comparison to day 1. In the MWM, independent t‐test revealed significant difference (*p* < 0.01) in the latency to reach the platform on day 8 between sham and SAH mice, indicating the spatial learning and memory impairment in SAH mice (**Figure** **6**b). We also analyzed the swim speed to assess the potential impact of motor function as a confounding variable. Our evaluation revealed no significant difference in swim speed between the sham and SAH groups, indicating that motor performance did not contribute to the observed cognitive deficits (Figure 6c). Furthermore, the outcome of the probe trial showed a loss in spatial memory retention in SAH mice compared to sham animals, as evidenced by the significantly lower time spent by SAH mice in the target quadrant compared to the sham group. (Figure 6d).

The Y‐maze test was performed to test the cognitive function that assesses the natural exploration behavior of rodents. Our results indicated that SAH led to memory impairment in mice compared to sham controls, as evidenced by significantly (*p* < 0.001) less time spent by SAH mice in the novel arm compared to sham‐operated mice (**Figure** **7**a,b).

During the NOR test conducted to assess recognition memory in mice (Figure 7c,d), the sham‐operated mice exhibited a notably higher propensity for exploring novel objects in comparison to familiar ones, indicating an absence of memory impairment attributable to the surgical procedures. In contrast, the mice subjected to SAH displayed a significantly reduced exploration ratio for novel objects relative to familiar object (*p* < 0.01) in comparison to the sham‐operated mice, signifying cognitive impairment subsequent to SAH. Moreover, no significant difference was observed in the exploration ratio for novel and familiar objects in SAH mice.

Next, we performed the FC test, the aversive associative learning and memory assessment of sham and SAH‐operated mice (Figure 7e,f). The test concluded impaired tone and contextual conditioning in SAH animals. SAH mice showed significantly reduced percent freezing compared to sham controls in these tests, indicating an injury‐induced impairment of amygdala‐ and hippocampus‐dependent memory function.

## 4 Discussion

Long‐term neurocognitive deficits are the primary driver of poor quality of life and low rate of return to work in patients who suffer SAH. New treatment strategies to combat the pathophysiological contributors to these long‐term cognitive deficits due to EBI and DCI are desperately needed, and animal models that more fully recapitulate the pathology and disability seen in SAH patients would aid investigators’ efforts. To date, the lack of a mouse model of SAH that consistently and reproducibly causes trackable long‐term neurocognitive deficits across independent laboratories has been a barrier to the identification of novel, effective, and translatable therapies. The rich toolset of genetically modified mice makes this species particularly attractive for such studies.

The variability in achieving SAH through the well‐known endovascular perforation technique renders it less controllable compared to the direct injection of a specific blood volume into the subarachnoid cisterns. This particular method is utilized for the single‐injection prechiasmatic model, the cisterna magna injection model, and the double‐injection prechiasmatic model introduced in our paper. The observed variability in SAH associated with the endovascular perforation model is the most plausible reason for differing results related to long‐term neurocognitive deficits.

In the present work, we sought to improve SAH disease modeling by modifying the previously reported pre‐chiasmatic injection mouse model. The principal modification involved administering heterologous blood into the prechiasmatic cistern over two days, with the second blood injection performed 24 h after the first injection. Thirty days after 2^nd^ injection, we conducted a series of neurobehavioral assessments and observed impaired cognitive functions in SAH mice compared to the sham group. These impairments include deficits in spatial learning and memory functions reliant on the hippocampus, novel object recognition associated with the cortical areas, and fear conditioning processes linked to the amygdala. Notably, these deficits displayed a significant association with EBI, as confirmed by cerebral edema noted on MRI. Furthermore, we demonstrated that the same brain regions exhibited several SAH‐related pathologies, including microthrombosis, microgliosis, astrocytosis, and apoptosis. Conversely, long‐term neurocognitive deficits were not observed in a single blood injection mouse model of SAH (Figure S1, Supporting Information), which aligns with previous experimental SAH studies.^[^ ^22^, ^43^ ^]^

Neuroinflammation and microvascular thrombosis have both been linked to the pathophysiology of brain injury following SAH in patients.^[^ ^44^, ^45^ ^]^ Similarly, in our mouse model of SAH, we observed significant chronic neuroinflammation, indicated by sustained microgliosis and astrocytosis by histology. Astrocytes have been found to perpetuate BBB impairment, glymphatic–lymphatic system dysfunction, reactive oxygen species generation, and cell death after SAH.^[^ ^46^ ^]^ We also found evidence of microvascular thrombosis at later time points after induction of SAH in our mouse model. Our findings underscore the significance of our prechiasmatic double injection mouse model of SAH in helping to identify and potentially address the underlying mechanisms of SAH‐induced neurocognitive dysfunction. Our findings suggest that ongoing astrocytosis and microgliosis, along with the late effects of microvascular thrombosis, are likely, at least in part, part of the complex array of pathophysiological events following SAH that ultimately lead to enduring neurocognitive deficits. This double injection mouse model of SAH appears to provide a reliable metric of disease severity, wherein the magnitude of SAH, moderated by blood volume, correlates with the pathophysiological mechanisms induced by SAH and its long‐lasting neurocognitive repercussions.

In contrast to available direct injection and endovascular perforation mouse models of SAH, our prechiasmatic double blood injection mouse model of SAH successfully and reproducibly detect long‐term cognitive and functional deficits. This appears to be an optimal approach for studying the severe long‐term clinical consequences of delayed neurological deterioration in human SAH. Important advantages of our mouse model of SAH compared with existing methods are summarized in Table 1.

Our protocol presents a promising approach to conducting a more comprehensive and reliable study. Therefore, successful implementation of this protocol has the potential to significantly advance how we approach the study and treatment of SAH and represents a notable improvement for the field.

## 5 Conclusion 

The proposed prechiasmatic double injection mouse model of SAH offers a promising means of testing genetically modified strains with ease of application, reliability, and high spatiotemporal control over the injected blood volume. This approach enables fair comparisons between animals and experimental groups by reducing experimental variability. A standardized preclinical SAH model of this nature would be optimal for studying the pathophysiology of SAH and evaluating potential therapeutic avenues to improve outcomes. The implementation of such a model would, therefore, provide a valuable translational tool for advancing our understanding of SAH and its treatment.

## Conflict of Interest

The authors declare no conflict of interest.

## Ethics Declarations

Ethics approval and consent to participate: This study was performed under an institutionally approved animal protocol and in accordance with the guidelines of the National Institutes of Health.

## Author Contributions

D.D., J.M., and G.J.Z. contributed equally to this work and also shares senior authorship. D.D., J.M., and J.W.N. performed concept and design. D.D., J.M., J.W.N., J.Q., S.S., and S.C. performed experimentation. D.D., J.M., and J.W.N. performed acquisition, analysis, or interpretation. D.D. and G.J.Z. performed drafting of the article. D.D., J.M., J.W.N., and G.J.Z. performed critical revision of the article for important intellectual content. J.M. and G.J.Z. performed supervision. B.M. and A.M. performed Supporting. All authors have reviewed and approved the final version of the manuscript for submission.

## Supporting information



Supporting Information

## Data Availability

The data that support the findings of this study are available from the corresponding author upon reasonable request.
