# Supplementary material for: Development and Validation of a Prechiasmatic Mouse Model of Subarachnoid Hemorrhage to Measure Long‐Term Cognitive Deficits
Source: Adv Sci (Weinh). 2024 Oct 23;11(46):2403977. doi: 10.1002/advs.202403977 (PMC11633547; doi:10.1002/advs.202403977)

**Figure S1:** **Learning and memory in single injection prechiasmatic mouse model of SAH.** WT mice underwent SAH or sham surgery and were evaluated 30 d post-SAH for cognitive function. Measurement of mean escape latency in the Morris water maze visible platform test (**a**). Time to reach a visible platform was quantified during cued training over 9 days (**b**). Novel arm entries in the Y-maze test (**c**). Exploratory ratio for familiar and novel objects (**d**)**.** Data show means ± SD. Student's t-test. vs. Sham group. N = 5-11 per group.


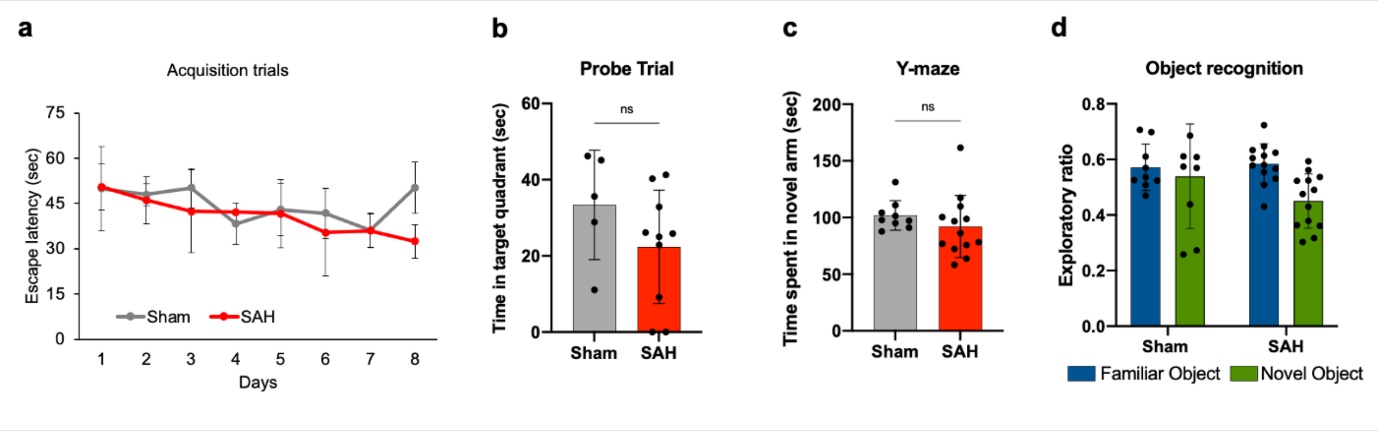

Supplement: Supplementary file 1 — Supporting Information [file ADVS-11-2403977-s001.docx]
